# Bladder filling in patients undergoing prostate radiotherapy on a MR-linac: The dosimetric impact

**DOI:** 10.1016/j.tipsro.2022.02.002

**Published:** 2022-02-16

**Authors:** Gillian Adair Smith, Alex Dunlop, Helen Barnes, Trina Herbert, Rebekah Lawes, Jonathan Mohajer, Alison C. Tree, Helen A. McNair

**Affiliations:** a Royal Marsden NHS Foundation Trust, United Kingdom; b Institute of Cancer Research, United Kingdom

**Keywords:** MR-linac, Prostate, Radiotherapy, Bladder filling

## Abstract

The implementation of adaptive radiotherapy for prostate cancer compensates for inter-fraction motion, at the penalty of increased time in room. The subsequent increase in bladder filling may impact dosimetry, which we have investigated on ten patients treated on the MR-linac. Patients drank water before treatment, to achieve a bladder volume of 200–300 cm^3^. Bladder and bowel were re-outlined offline on 140 images and plans recalculated. All mandatory bladder dose constraints and 99.1% of bowel constraints were achieved at time of treatment, despite varying bladder volumes and varying adherence to original bladder filling guidance.

## Highlights

• Patient compliance with bladder filling varies.
• The majority of dose constraints can be achieved despite variations in bladder filling.
• For patients on the MR-linac, a small bladder at the start of the session does not indicate mandatory dose constraints will not be met at time of treatment.

## Introduction

Radiotherapy is an effective treatment of prostate cancer with up to 91% of patients being biochemical or clinical failure-free after five years [1]. With improved long-term survival, minimising toxicity and reducing side effects is important. Daily motion of the prostate and organs at risk (OAR) can increase the amount of normal tissue irradiated and hence increase side effects. Patient preparation guidance to reduce internal organ motion include rectal stabilising methods, for example maintaining an empty rectum by using enemas [2], and treating with a full bladder to exclude bladder wall and move small bowel away from the high dose region [3], [4], [5]. To maintain a full bladder requires patients to drink a predefined amount of water at a particular time prior to treatment (wait time) which can increase the time spent in the department and may cause anxiety.

Adaptive radiotherapy (ART) has the potential to compensate for inter-fraction motion by enabling daily online re-contouring and re-optimisation and is now clinically available [6], [7], [8]. However, reported treatment workflow times of between 35 and 60 min could potentially impact treatment accuracy. The increase in time could allow bladder filling and cause intra-fraction prostate motion. Prior to implementing treatment on the Elekta Unity MR-linac (Elekta AB, Stockholm, Sweden) we estimated a workflow time of 45 mins. The conventional guidance for bladder filling was subsequently reduced. The aim was to ensure patients had a sufficiently full bladder at the time of treatment delivery, whilst being able to hold their bladder for the duration of treatment. However, with the possibility that adaptive radiotherapy may mitigate the need for bladder filling, an audit was undertaken to establish the importance of adhering to strict filling guidelines. We present the evaluation of the effectiveness of the revised guidance on bladder volume reproducibility and dose constraints.

## Materials and methods

The first ten patients recruited to the PRISM trial (Prostate Radiotherapy Integrated with Simultaneous MRI, NCT03658525) were included. Patients were asked to empty and then drink 350 ml water with a 45 mins (planning CT;pCT) and 30 mins (treatment) wait, to achieve a bladder volume of 200–300 cm^3^. Enemas were prescribed for two days prior to the pCT and start of radiotherapy, and for the first 10 fractions of treatment. The treatment planning process was performed as described previously [8]. Patients were treated with 60 Gy in 20 fractions using the Adapt to Shape (ATS) workflow on the Elekta Unity MR-linac (Elekta AB, Stockholm, Sweden). A T2-weighted MRI (session image) was acquired and rigidly registered to the reference image (pCT or pMRI). Propagated contours (target and OARS) from the reference image were amended as necessary by a clinical oncologist. After optimisation based on the amended contours, a second T2-weighted MRI (verification image) was acquired and registered with the session image to assess intra-fraction motion. A subsequent Adapt to Position (ATP) of the ATS plan was performed when necessary [8].

### Bladder filling protocol

Variations to volume of water and wait time were permissible to meet optimal bladder volume and provide patient comfort. To establish adherence to bladder filling protocols, volume of water drank and wait time was recorded daily using MOSAIQ (Elekta AB, Stockholm, Sweden).

### Intra-fraction filling and OAR dose constraints

Session and verification images for each patient from the first three fractions, then weekly until the final fraction were analysed. These seven fractions were chosen as a purposeful sample to represent any variation during the treatment course [9].

**Intra-fraction filling:** Whole bladder and bowel loops, up to 2 cm from PTV, were re-outlined offline for each fraction listed above on both the session and verification images by one radiographer using Monaco TPS (version 5.4, Elekta AB, Stockholm, Sweden). The volume of the bladder and time of MRI acquisition was documented. The mean rate of intrafraction filling for each patient was determined.
**OAR dose constraints:** The dose to the bladder and bowel was determined by re-calculating the clinically delivered plan on the re-contoured session and verification image offline. No other amendments were made to the plan. The daily fraction dose calculations scaled to the full prescription, i.e. 20 fractions, are reported.

**Analysis**: Adherence to drinking instructions was defined as following the drinking protocol (350 ml, 30 mins) for the majority of treatment (60%). Patients were grouped by those who drank less, exact, or more than the bladder filling protocol suggests. The difference in bladder volume and filling rate between groups was compared using Kruskal-Wallis test for non-parametric data. The number of mandatory and optimal dose constraints met, to the bladder and bowel, were determined.

Patients in a patient and public involvement group (PPI), treated on the MR-linac for prostate cancer were asked about the impact of bladder filling using four questions:

1. What impact would it have had on your radiotherapy experience to not fill your bladder each day?
2. If there were no bladder filling requirements, what impact would it have had on your day spending less time in the department?
3. How did having a full bladder affect you before treatment?
4. How did having a full bladder affect you during treatment?

## Results

### Bladder filling protocol

Volume of water and wait time was documented for 192 of 200 fractions. Bladder filling guidelines were followed on 91 occasions. For the remaining 101 fractions, volume of water and/or wait time was altered to meet optimal bladder volume or provide patient comfort. The volume of water varied between 175 ml and 525 ml and the wait time from 15 min to 60 min. There were six occasions when patients could not hold their bladder for the duration of treatment (patient 1, n = 1; patient 5, n = 5).

### Intra-fraction filling and OAR dose constraints

In total, 140 images were re-contoured and available for analysis (70 session and 70 verification).

**Intrafraction filling:** The median (range) bladder volume at session image was 121 cm^3^ (46–708 cm^3^), Fig. 1a. The median (range) bladder volume at verification image, was 211 cm^3^ (70–933 cm^3^), Fig. 1b. The median (range) time between the session and verification images was 25 min (19–34 min)**.**

**Fig. 1a:**
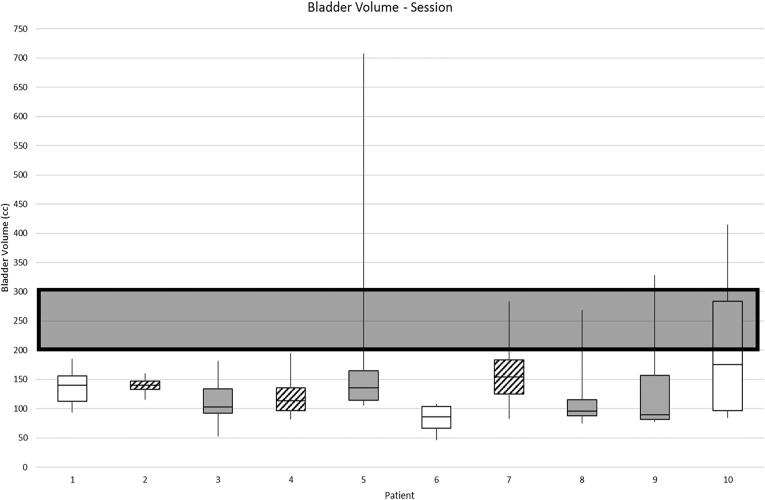
Absolute bladder volume at session image (grey box indicates preferred volume as per PRISM).

**Fig. 1b:**
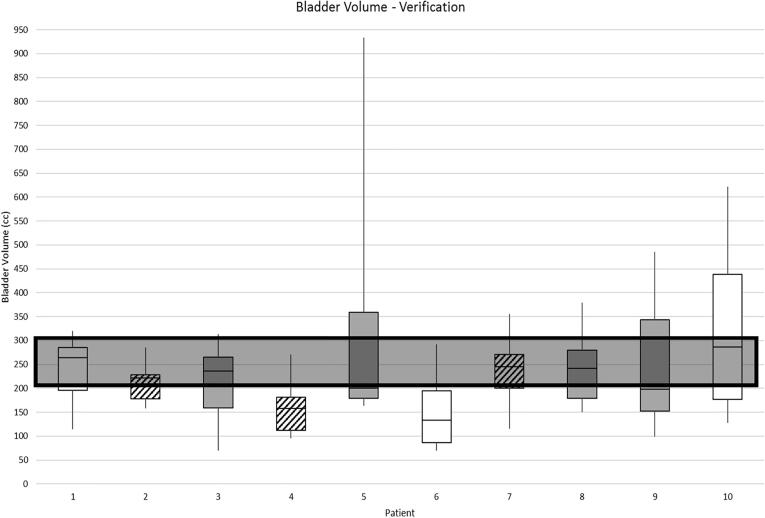
Absolute bladder volume at verification image (grey box indicates preferred volume as per PRISM).

Key

**Table:** 

		Drank more than recommended by bladder filling guidelines for over 60% of fractions
		Drank exactly as stated in bladder filling guidelines
		Drank less than recommended by bladder filling guidelines for over 60% of fractions

There was no statistical difference in absolute bladder volumes at verification scan between patients who drank more or less than recommended for the majority of treatments, compared with those who drank exactly as recommended.

Large bladder volumes (708 cm^3^ at session and 933 cm^3^ at verification) were observed in one patient who waited 10 mins longer than usual. The fill rate in this fraction was 9.82 cm^3^ per minute, the second largest fill rate. The greatest fill rate was observed by patient 8 (9.88 cm^3^ per minute) when the bladder filled from 74 cm^3^ to 292 cm^3^.

**Fig. 2:**
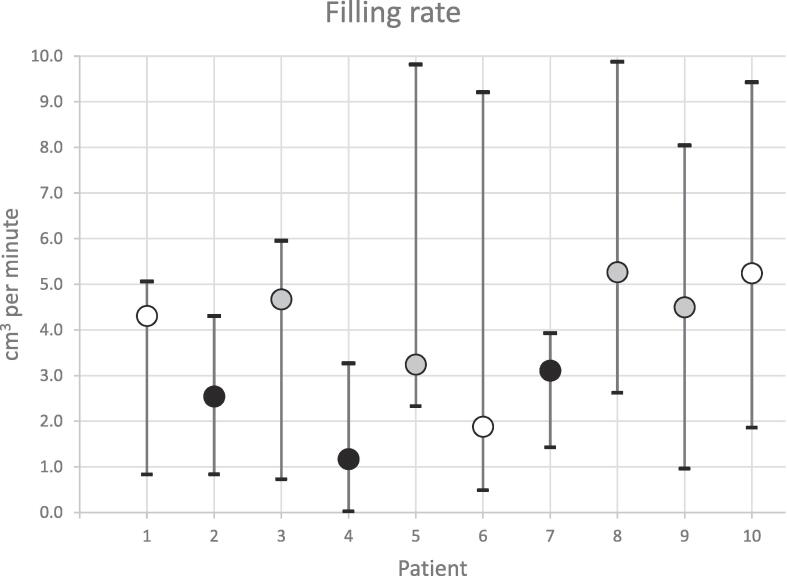
Intra-fraction bladder filling rate median (range).

The median (range) rate of bladder filling over all fractions was 3.34 cm^3^ per minute (0.03–9.88 cm^3^), Fig. 2. The range of median fill rates per patient was 1.17–5.27 cm^3^ per minute. Less than half (4/10) of patients drank as required for the majority of treatment. Patients who drank more than the recommended guidelines (n = 3) had a significantly slower rate of bladder filling (*p = <0.001*).

Key

**Table:** 

		Drank more than recommended by bladder filling guidelines for over 60% of fractions
		Drank exactly as stated in bladder filling guidelines
		Drank less than recommended by bladder filling guidelines for over 60% of fractions

**OAR dose constraints:** All mandatory bladder dose constraints were met on the recalculated plans on both the session and verification image (Table 1). Optimal bladder dose constraints missed on the session image occurred when the bladder volume was <200 cm^3^. The number of optimal dose constraints missed on the verification image was reduced and were mainly where bladder volumes were <200 cm^3^ (n = 41) or between 200 cm^3^ to 300 cm^3^ (n = 15) with only one case where the bladder volume was >300 cm^3^.

**Table 1:** Number of bladder and bowel clinical goals not satisfied for session image and verification image patient representations. Patients are grouped by bladder volume at each timepoint, with 200–300 cm^3^ the PRISM recommended bladder volume. PRISM mandatory clinical goals are denoted by †, remaining clinical goals optimal.

**OAR**	**Clinical goal**	**Session image**			**Verification image**		
		<200 ml	200 ml < 300 ml	>300 ml	<200 ml	200 ml < 300 ml	>300 ml
		(n = 63)	(n = 3)	(n = 4)	(n = 34)	(n = 24)	(n = 12)
Bladder	V40.5 Gy < 50%	4	0	0	1	0	0
	V48.7 Gy < 25%	10	0	0	9	0	0
	V52.7 Gy < 50%	0	0	0	0	0	0
	V56.76 Gy < 35%	53	0	0	28	15	1
	V60.8 Gy < 25%	2	0	0	3	0	0
	V56.76 Gy < 5% †	0	0	0	0	0	0
	V60.8 Gy < 3% †	0	0	0	0	0	0

Bowel	V36.5 Gy < 78 cc	0	0	0	0	0	0
	V40.5 Gy < 17 cc	0	0	0	0	0	0
	V44.6 Gy < 14 cc	0	0	0	0	0	0
	V48.7 Gy < 0.5 cc	11	0	0	3	0	0
	V36.5 Gy < 158 cc †	0	0	0	0	0	0
	V40.5 Gy < 110 cc †	0	0	0	0	0	0
	V44.6 Gy < 28 cc †	0	0	0	0	0	0
	V48.7 Gy < 6 cc †	0	0	0	0	0	0
	V52.7 Gy < 0.01 cc †	16	1	0	3	0	0

A similar pattern was observed with the bowel dose constraints, with most dose constraints missed in the bladder <200 cm^3^ (n = 27) group and only one where the bladder volume was between 200 cm^3^ to 300 cm^3^. 0.9% of mandatory and 1% of optimal dose constraints were missed on the verification image. All constraints missed in the verification image were where the bladder volume was <200 cm^3^ and occurred in two patients. In one patient the bowel was abutting the seminal vesicles and intra-fraction bladder filling had little impact. In the other, a change in patient position increased the bowel volume within the PTV and subsequently increased the dose.

**PPI Group feedback**: Of the two patient responses, the negative comments were regarding the full bladder making treatment uncomfortable. There were no issues prior to treatment although one patient would have liked to spend less time on the department. The impact on treatment included comments such as:

‘Challenges in holding my full bladder during the treatment process’.

‘Initially I drank too much and too soon before treatment, this caused some anxious moments’

Both patients responded that not filling bladder would have made been a more positive experience.

‘By not having to fill my bladder each day would have given me a more positive experience’

‘It would have made the treatment more comfortable and me less anxious’

## Discussion

We have shown that mandatory dose constraints can be achieved despite varying bladder volumes and varying adherence to a bladder filling protocol. This suggests that strict adherence to bladder filling protocols is not essential to meet clinical dose constraints on the MR-linac.

Although adaptive radiotherapy can compensate for inter-fraction changes, the anatomical impact of a full bladder displacing the bowel remains an advantage. More constraints were met on the verification images with a larger bladder volume (median 211 cm^3^). Therefore, the aim for patients to be treated with a ‘non-empty’ bladder remains. Of the three instances where mandatory bowel constraints were not met at time of treatment, bladder filling did not improve the plan. Both patients in these three instances drank more than the recommended guidelines. Interestingly, both patients had a slower rate of bladder filling despite increasing the volume drank/wait time (patient 2, 2.5 cm^3^ per minute/40 mins wait time daily; patient 4, 1.2 cm ^3^ per minute/525 ml cups daily). It could be postulated that these patients were dehydrated. Increasing hydration prior to treatment may assist in achieving an acceptable bladder volume but should be approached cautiously. Pre-hydrating by drinking 1–2 L a day for 3 consecutive days has been found to produce extremely large bladder volumes that were not reproducible at treatment [10].

The bladder filling rate was variable with a large range over all fractions (9.85 cm^3^ per minute), indicating that one protocol may not be suitable for all patients. Similar fill rates have been identified in other populations of 4.6 ± 2.9 min^−1^ [11]. The variation in bladder filling in this study, the observation that fill rate decreases during treatment course [11], and the variation of bladder filling protocols in many centres[12] suggest that more flexible protocols could be implemented for patients treated on all radiotherapy platforms. For patients on the MR-linac, if a small bladder is seen on the session image, we could anticipate that by the time of the verification image mandatory dose constraints will likely be met, therefore there is no need to ask the patient to wait and fill their bladder. It is acknowledged that optimisation for a smaller bladder volume than that anticipated at the time of treatment may result in unwarranted compromise to other aspects of plan quality, such as conformality or dose to other OARs.

Limitations of the study include a small sample size. The audit took place over eight months, however the total number of patients treated during that time was small. One protocol is unlikely to be appropriate for all patients because there are many aspects which affect bladder filling such as general hydration, co-morbidities, and time of day. Whether bladder volumes fill at a consistent rate or plateau has not been addressed in this study and would require bladder volumes from an additional time point, for example post treatment. Although strong conclusions cannot be drawn from the data there are indications for further study.

Simplifying bladder preparation has the additional potential to positively impact patient experience by reducing stress and anxiety associated with achieving and maintaining the correct bladder status for all treatment.

## Conclusions

All mandatory bladder dose constraints and 99.1% of mandatory bowel dose constraints were achieved, at time of beam-on, for patients receiving prostate radiotherapy on a MR-linac, despite varying bladder volumes and varying adherence to a bladder filling protocol. A small bladder volume on a session image does not indicate that mandatory dose constraints will not be met at time of treatment. Further investigation into bladder filling protocols may improve patient experience.

## Declaration of Competing Interest

The authors declare the following financial interests/personal relationships which may be considered as potential competing interests: This report is independent research supported by the National Institute for Health Research and Health Education England (HEE/ NIHR ICA Programme Senior Clinical Lectureship, Dr Helen McNair, ICA-SCL-2018-04-ST2-002). The views expressed in this publication are those of the author(s) and not necessarily those of the NHS, the National Institute for Health Research or the Department of Health and Social Care. We also acknowledge NHS funding to the NIHR Biomedical Research Centre at The Royal Marsden and The Institute of Cancer Research. AT acknowledges support from The Rosetrees Trust. The Institute of Cancer Research is supported by Cancer Research UK Programme Grants (C33589/A19727, C33589/A19908 and C33589/A28284). ICR/RMH is a member of the Elekta MR Linac Research Consortium and receives institutional support from Elekta. Dr. Tree reports grants and other from Elekta, during the conduct of the study; grants from Accuray, grants from Varian, other from Genesis Healthcare, outside the submitted work.
